# Interaction between Emotion and Attention Systems

**DOI:** 10.3389/fnins.2012.00139

**Published:** 2012-09-25

**Authors:** Shuhei Yamaguchi, Keiichi Onoda

**Affiliations:** ^1^Department of Neurology, Shimane UniversityIzumo, Japan

Rapid and efficient selection of emotionally salient or goal-relevant environmental stimuli is crucial for flexible and adaptive behaviors. Emotional events rapidly and automatically capture attention by activating subcortical neural structures (Ohman, 2005; Zikopoulos and Barbas, 2012). Behavioral data suggest that emotion processing, selective attention, and the interactions between them extract emotional values from sensory stimuli and lead to the generation of appropriate responses. The amygdala plays a pivotal role in emotional information processing. Furthermore, through feedback connections to sensory cortical processing regions, the amygdala may facilitate attentional, and perceptual processes. Thus, the link and interaction between emotion and attention systems have been the subjects of great interest in the field (Pessoa, 2005; Pourtois et al., 2012).

With regard to the interaction of emotion and attention, there are unsolved and controversial issues. For example, to what extent does emotional stimulus processing depend on attentional resource availability? Previous studies have shown conflicting results, probably due to several factors, including attentional load allocated to competing tasks. Some studies have demonstrated that emotional faces evoke amygdalar responses even when attention is diverted to other stimuli, suggesting that some types of emotional perception occur outside of top-down directed attention (Vuilleumier et al., 2001; Anderson et al., 2003). However, others have suggested that the perception of emotional items requires attention. This was demonstrated with attentional manipulations that were designed to utilize a high level of processing resources, leaving less bandwidth for processing unattended emotional items (Bishop et al., 2007; Lim et al., 2008). Another conflicting issue is whether the spatial location of emotional stimuli (foveal or peripheral) affects emotion processing. Previous studies have demonstrated that the amygdala was activated independent of attentional location when emotional stimuli were presented in the peripheral visual field (Williams et al., 2005); however, this was not the case when stimuli were presented foveally. Ignored emotional faces presented foveally led to increased amygdalar response only under low attentional load conditions (Pessoa, 2005). This discrepancy in the interaction between attentional load and stimulus location may be related to the anatomical differences in visual pathways for foveal and peripheral stimuli (i.e., parvocellular and magnocellular pathways, respectively).

Morawetz et al. (2010) directly addressed these questions in a series of block-designed functional magnetic resonance imaging (fMRI) experiments, in which they systematically manipulated both the attentional load and the spatial position of emotional stimuli. They imposed four types of tasks with two attentional conditions; two *attend-faces* conditions (gender-matching and emotion-matching tasks) and two *ignore-faces* conditions (easy and difficult digit/letter-matching tasks). The behavioral data suggested that the experimental design successfully controlled the attentional load and perceptual properties of emotional stimuli (i.e., image size) presented foveally or peripherally in the visual field. Their study resulted in two main findings. First, amygdalar activity was strongly suppressed during high attentional loads, which suggests that emotional information processing might be suspended when attention-demanding cognitive tasks are performed simultaneously. Although this finding is not new (Pessoa, 2005), this was the first study in which attentional load was systematically manipulated in a quantitative manner. The result expands the hypothesis that amygdalar emotional information processing is dependent on available attentional resources and could be affected by top-down attentional control from other brain structures. Second, the eccentricity of the emotional stimuli did not affect the amygdalar response, and there was no interaction effect between attentional load and stimulus location. This finding did not support the hypothesis of predominant processing of peripherally presented emotional stimuli, but it is hard to compare the results with those of previous studies because the experimental designs and analysis methods were different. The block-design paradigm and region of interest–based analysis employed in the current experiment may have reduced the sensitivity for detecting habituated and/or very focal amygdalar responses.

In summary, Morawetz et al. (2010) provided compelling evidence of a spatial attention effect on emotion processing in the amygdala by using a well-designed experimental paradigm. A recent fMRI study with a cue-associated covert attention task reported results in line with their findings (Brassen et al., 2010). However, their analyses of fMRI data were restricted to the amygdala and fusiform gyrus. It is obvious that spatial attention involves a widely distributed network, including the prefrontal cortex, thalamus, parietal cortex, and subcortical structures. Studies have suggested that emotionally salient and threatening stimuli automatically capture attention (Carlson et al., 2009). Thus, further analyses of blood oxygen level–dependent signals in other brain areas engaged in attentional processes would provide new insight into the bidirectional interaction between attention and emotion systems. Because the processing speeds of both networks may be too rapid to detect with fMRI, other neurophysiological tools, such as electroencephalography or magneto encephalography, could be suitable assessments (Pessoa, 2010). The functional interaction of emotional and attentional systems remains to be fully explored, but it might play an important role in psychopathological conditions, such as anxiety or phobia.
